# Genomic surveillance of antimicrobial resistant bacterial colonisation and infection in intensive care patients

**DOI:** 10.1186/s12879-021-06386-z

**Published:** 2021-07-14

**Authors:** Kelly L. Wyres, Jane Hawkey, Mirianne Mirčeta, Louise M. Judd, Ryan R. Wick, Claire L. Gorrie, Nigel F. Pratt, Jill S. Garlick, Kerrie M. Watson, David V. Pilcher, Steve A. McGloughlin, Iain J. Abbott, Nenad Macesic, Denis W. Spelman, Adam W. J. Jenney, Kathryn E. Holt

**Affiliations:** 1 Department of Infectious Diseases, Central Clinical School, Monash University, Melbourne, Victoria Australia; 2 Microbiology Unit, Alfred Health, Melbourne, Victoria Australia; 3 Department of Microbiology and Immunology, University of Melbourne, Melbourne, Victoria Australia; 4 Infectious Diseases Clinical Research Unit, The Alfred Hospital, Melbourne, Victoria Australia; 5 Intensive Care Unit, The Alfred Hospital, Melbourne, Victoria Australia; 6 Australian and New Zealand Intensive Care – Research Centre, School of Public Health and Preventive Medicine, Monash University, Melbourne, Victoria Australia; 7 Department of Infectious Diseases, The Alfred Hospital, Melbourne, Victoria Australia; 8 London School of Hygiene and Tropical Medicine, London, UK

**Keywords:** Antimicrobial resistance (AMR), Colonisation, Transmission, Genomic surveillance, Intensive care

## Abstract

**Background:**

Third-generation cephalosporin-resistant Gram-negatives (3GCR-GN) and vancomycin-resistant enterococci (VRE) are common causes of multi-drug resistant healthcare-associated infections, for which gut colonisation is considered a prerequisite. However, there remains a key knowledge gap about colonisation and infection dynamics in high-risk settings such as the intensive care unit (ICU), thus hampering infection prevention efforts.

**Methods:**

We performed a three-month prospective genomic survey of infecting and gut-colonising 3GCR-GN and VRE among patients admitted to an Australian ICU. Bacteria were isolated from rectal swabs (*n* = 287 and *n* = 103 patients ≤2 and > 2 days from admission, respectively) and diagnostic clinical specimens between Dec 2013 and March 2014. Isolates were subjected to Illumina whole-genome sequencing (*n* = 127 3GCR-GN, *n* = 41 VRE). Multi-locus sequence types (STs) and antimicrobial resistance determinants were identified from de novo assemblies. Twenty-three isolates were selected for sequencing on the Oxford Nanopore MinION device to generate completed reference genomes (one for each ST isolated from ≥2 patients). Single nucleotide variants (SNVs) were identified by read mapping and variant calling against these references.

**Results:**

Among 287 patients screened on admission, 17.4 and 8.4% were colonised by 3GCR-GN and VRE, respectively. *Escherichia coli* was the most common species (*n* = 36 episodes, 58.1%) and the most common cause of 3GCR-GN infection. Only two VRE infections were identified. The rate of infection among patients colonised with *E. coli* was low, but higher than those who were not colonised on admission (*n* = 2/33, 6% vs *n* = 4/254, 2%, respectively, *p* = 0.3). While few patients were colonised with 3GCR- *Klebsiella pneumoniae* or *Pseudomonas aeruginosa* on admission (*n* = 4), all such patients developed infections with the colonising strain. Genomic analyses revealed 10 putative nosocomial transmission clusters (≤20 SNVs for 3GCR-GN, ≤3 SNVs for VRE): four VRE, six 3GCR-GN*,* with epidemiologically linked clusters accounting for 21 and 6% of episodes, respectively (OR 4.3, *p* = 0.02).

**Conclusions:**

3GCR-*E. coli* and VRE were the most common gut colonisers. *E. coli* was the most common cause of 3GCR-GN infection, but other 3GCR-GN species showed greater risk for infection in colonised patients. Larger studies are warranted to elucidate the relative risks of different colonisers and guide the use of screening in ICU infection control.

**Supplementary Information:**

The online version contains supplementary material available at 10.1186/s12879-021-06386-z.

## Introduction

Healthcare associated infections (HAI) result in considerable morbidity and mortality, with the total burden in high income countries exceeding that of influenza, tuberculosis and other major communicable diseases combined [1]. The emergence and spread of antimicrobial resistant (AMR) organisms, including difficult-to-treat multi-drug resistant (MDR) strains, has further exacerbated this problem [2], and as a consequence several MDR healthcare-associated bacterial pathogens are now recognised by the World Health Organization (WHO) as urgent threats to public health [3]. Among the WHO’s top priorities are carbapenem-resistant Gram-negatives (GNs, specifically *Enterobacteriaceae*, *Acinetobacter baumannii* and *Pseudomonas aeruginosa*) as well as vancomycin-resistant enterococci (VRE).

In Australia, it is estimated that one in ten hospitalised patients suffers an HAI, and the most common MDR organisms are VRE, methicillin-resistant *Staphylococcus aureus* and extended-spectrum beta-lactamase (ESBL) producing *Enterobacteriaceae* [4]. Fortunately carbapenem-resistance has so far remained rare, although the prevalence has been slowly increasing [5]. Notably, Australia suffers a particularly high rate of vancomycin resistance among enterococcal infections (~ 45% of *Enterococcus faecium* bacteremias [5]), primarily due to the endemic spread of healthcare-associated *E. faecium* sequence types (STs) 17, ST80 and ST796 [5–8].

Asymptomatic gut colonisation is considered a key risk factor for enterococcal, *Enterobacteriaceae* and other GN infections [9–13] and in the case of VRE has been associated with longer ICU stay and treatment costs [12, 14]. A number of hospitals have implemented rectal screening programs to identify patients colonised with VRE and/or carbapenemase-producing *Enterobacteriaceae* in high-risk wards such as intensive care units (ICUs), oncology and hematology wards [7, 8, 15, 16]. However, few facilities conduct routine screening for ESBL-*Enterobacteriaceae* or other high-risk carbapenem-susceptible GNs in part due to a lack of evidence to inform the interpretation of these results and to appropriately target infection control measures. For example, only a small number of studies have investigated the risks associated with ESBL-*Enterobacteriaceae* colonisation at a species-specific level [9, 11, 12, 17–20], but there is emerging evidence to suggest important variation, both in terms of the risk of infection and the risk of transmission in the hospital setting. We propose that a better understanding of these risks, as well as the broader colonisation and infection dynamics of AMR organisms in these settings, will facilitate better targeting of infection prevention and control practices.

Here we report a genome-resolved snapshot of high-risk AMR bacteria from rectal swabs and clinical specimens collected in an Australian ICU over a three-month period. We focus on GN organisms resistant to third-generation cephalosporins (including ESBL-*Enterobacteriaceae*) in addition to VRE*.* We leverage the genome data to perform high-resolution analysis of these bacterial populations to; i) compare infecting and colonising organisms isolated form the same patient; ii) characterise population diversity at the strain level; and iii) identify putative nosocomial transmission clusters.

## Methods

### Recruitment and specimen collection

The Alfred Hospital, Melbourne, Australia is a 350-bed tertiary referral hospital including a 45-bed ICU providing care for general medical and surgical patients plus specialist cardiac and trauma services. We conducted a prospective surveillance study for rectal colonisation of third generation cephalosporin-resistant GN (3GCR-GN) organisms and VRE in patients admitted to the ICU from 21 Dec 2013 to 7 April 2014. This study was approved by the Alfred Health Human Research Ethics Committee, Project numbers #550/12 (19 February 2013) and #526/13 (10 December 2013). Patients aged ≥18 years and considered by nursing staff as likely to remain in the ICU > 2 days were eligible for inclusion. The requirement for informed consent to participate was waived by the same ethics committee (Alfred Health Human Research Ethics Committee) as screening for AMR organisms is considered an infection control and surveillance activity, however patients were given the option to refuse screening. Rectal swabs were collected at time of recruitment (day 0–2 of ICU admission) and subsequently each 5–7 days during ICU stay. Information on age, sex, dates of hospital and ICU admission/s, surgery in the last 30 days, and antibiotic treatment in the last 7 days were extracted from hospital records when swabs were taken. Dates of discharge and/or death were extracted from hospital records at the conclusion of the study. All clinical isolates recovered from ICU patients and identified as 3GCR-GN or VRE by the diagnostic laboratory as part of routine care were also stored for inclusion.

### Bacterial culture and sequencing

Gut colonising 3GCR-GN and VRE were identified by culture on ceftazidime and chromID VRE agar plates (BioMérieux, Marcy L’Etoile, France), respectively. Presumptive 3GCR-GN and VRE colonies were subjected to species identification via matrix-assisted laser desorption ionization time-of-flight analysis with a Vitek MS (BioMérieux). Clinical isolates were collected and identified via standard diagnostic protocols [10]. Genomic DNA was extracted and sequenced on the Illumina platform. Twenty-three isolates were selected for sequencing on the Oxford Nanopore MinION platform as described previously [21]. See Supplementary Methods, Additional File 1 for full details.

### Genomic analyses

Genomes were assembled using Unicycler v0.4.7 [22] and subjected to quality control (Supplementary Methods, Additional File 1). Species were identified using Kraken v1.0 [23] and multi-locus sequence types (STs) were identified from assemblies using mlst (github.com/tseemann/mlst) where schemes were available (accessed from pubmlst.org [24]).

AMR genes were identified from final assemblies using Kleborate (https://github.com/katholt/Kleborate) [25] which performs nucleotide and protein BLAST against a curated version of the CARD database, and results were interpreted in a species-specific manner (i.e. to distinguish intrinsic from acquired genes). These data were used to calculate the predicted number of acquired AMR classes per isolate (defined for each species as described in [26], Supplementary Methods, Additional File 1). MDR was defined as predicted acquired resistance for ≥3 drug classes [26].

Single nucleotide variants (SNVs) were identified by mapping Illumina reads to the relevant completed chromosomal reference genomes (ST-specific, Supplementary Table 1, Additional File 2) using Bowtie2 v2.2.9 [27] and SAMtools v1.9 [28], as implemented in the RedDog pipeline (github.com/katholt/RedDog) [29].

### Statistical analyses

Statistical analyses were performed using R v3.6.3 [30] and the tidyverse package [31]. Fisher’s Exact test and proportion test were used to test for differences in count data in two-by-two contingency tables (proportion test was applied where any single count was ≤5, otherwise Fisher’s Exact test was used). The Wilcoxon rank sum test was used to test for differences in age distributions, wherein the data did not fit a normal or other distribution appropriate for parametric testing. Univariate and multivariate logistic regression models were used to test risk factors for 3GCR-GN and VRE colonisation at ICU admission.

## Results

**Fig. 1:**
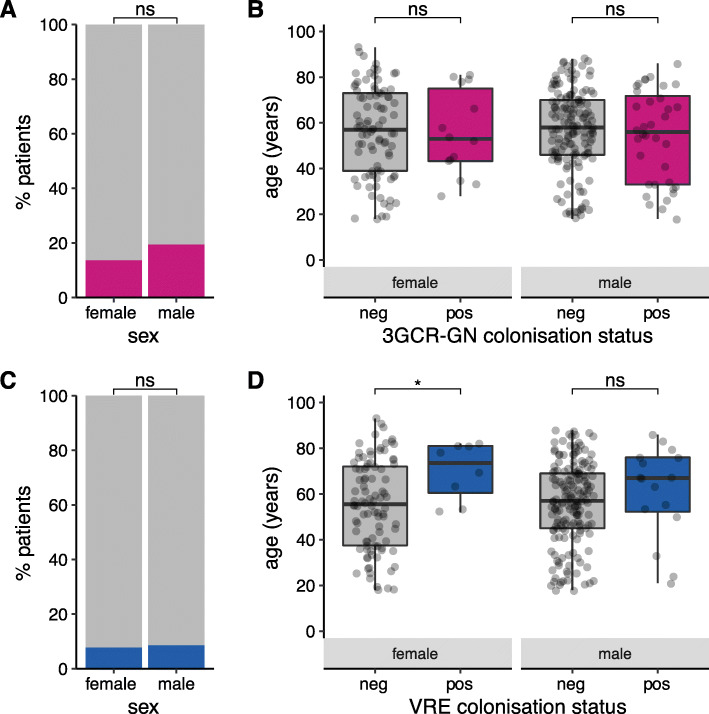
Prevalence of 3GCR-GN and VRE gut colonisation on admission to the ICU. Prevalence of third generation cephalosporin-resistant Gram-negative (3GCR-GN) organisms (**A**) and VRE (vancomycin-resistant enterococci) organisms (**C**) among males and females for whom rectal swabs were collected within two days of ICU admission. Age distributions are shown for culture-confirmed 3GCR-GN (**B**) and VRE (**D**) carriers and non-carriers, stratified by sex. ns, non-significant using Fisher’s Exact Test (**A, C**) or Wilcoxon Rank Sum test (**B, D**); *, *p* < 0.05

During the study period there were 716 patients admitted to the ICU, 31 (4.3%) patients had one or more 3GCR-GN infections (*n* = 41 isolates), and two (0.3%) had VRE infections (*n* = 2 isolates, both *E. faecium*, Fig. 1). Of 430 patients eligible for participation in rectal screening, 66 declined to participate and 311 contributed one or more rectal swabs (72.3% of eligible non-refusers). The majority of participants were swabbed within the first 2 days of ICU admission (baseline screening swabs, *n* = 287, 92.3%), including 79 with ≥1 additional follow-up swab (Fig. 1). Participants with baseline swabs were 64.5% male (*n* = 185), aged 18–93 years (median 57 years; IQR, 44–71 years, Supplementary Table 2, Additional File 1), and the majority (*n* = 218, 76.0%) were known to have had recent healthcare exposure prior to ICU admission (surgical procedure within the last 30 days, transferred from another ward with first admission > 2 days prior, or transferred from another hospital).

Third generation cephalosporin-resistant (3GCR) GN organisms were isolated from baseline screening swabs of 50 patients (17.4%, *n* = 56 isolates), while VRE were isolated from 24 (8.4%, *n* = 24 *E. faecium* isolates, no *E. faecalis* were identified; Supplementary Figure 1, Additional File 1). Co-colonisation with 3GCR-GN and VRE was identified in 12 patients (4.2%), indicating a significant association between these organisms (OR 5.6, 95% CI 2.2–15.5, *p* = 0.0001 using Fisher’s Exact Test). There were no significant differences in colonisation rates between males and females, and no association between age and colonisation status, with the exception of VRE in females (median age 73.5 years amongst carriers versus 55.5 years amongst non-carriers, *p* = 0.04 using Wilcoxon Rank Sum test; Fig. 1). Neither surgery within the last 30 days, recent healthcare exposure (defined as above), or antibiotic treatment within the last 7 days, were significantly associated with 3GCR-GN or VRE colonisation at baseline (using univariate and multivariate logistic regression, with age and sex as covariates; Supplementary Table 3, Additional File 1).

A total of 138 rectal swabs were collected from 103 patients between 3 days and 12 weeks after ICU admission (Supplementary Table 4, Supplementary Figure 2, Additional File 1). Twenty-five swabs (18.1%) from 18 patients (17.5%) were positive for ≥1 3GCR-GN organism (*n* = 30 isolates); 15 swabs (10.9%) from ten patients (9.7%) were positive for VRE (*n* = 15 isolates). Notably, 3GCR-GN organisms were cultured from 15/61 patients (24.6%) who were culture-negative for 3GCR-GN colonisation at baseline, and VRE were cultured from 13/75 (17.3%) patients who were culture-negative for VRE colonisation at baseline, consistent with acquisition and/or overgrowth of the organisms during ICU stay.

**Fig. 2:**
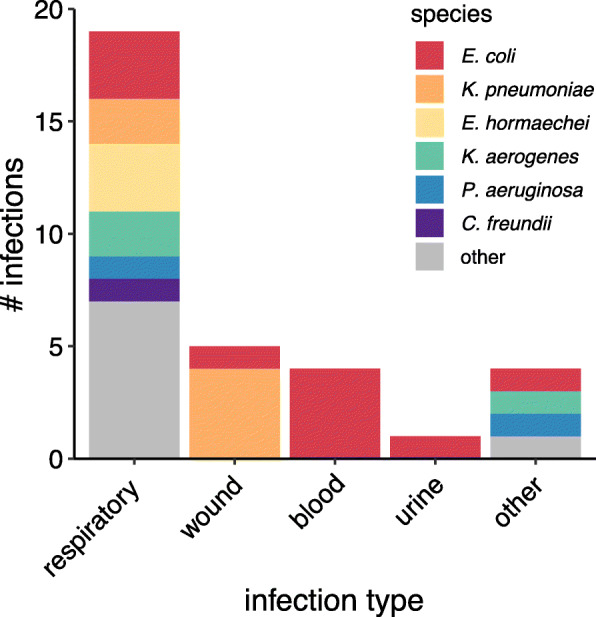
Infections caused by 3GCR-GN organisms. Bar chart shows the number of distinct respiratory, wound, blood, urine and other infections by species as indicated in the legend. Distinct infections were defined as those represented by unique combinations of species, multi-locus sequence type, patient and specimen type

Among the 127 3GCR-GN and 41 VRE isolates cultured from ICU patients, 112 (88.2%) and 41 (100%) respectively were successfully revived and sequenced (Supplementary Methods, Supplementary Figure 1, Additional File 1). We combined clinical and genome data to identify distinct colonisation and infection episodes, defined as unique combinations of species and ST (derived from whole-genome sequences (WGS)), per patient and specimen type. This identified 33 3GCR-GN and two VRE infection episodes (Table 1). The latter included one urinary tract infection for which the patient was prescribed amoxycillin (as recommended for treatment of VRE in the urinary tract, where a high concentration of amoxycillin can be achieved) and one respiratory infection treated with teicoplanin. 3GCR-GN infections were mainly respiratory (*n* = 19, 57.6%), wound (*n* = 5, 15.2%) and bloodstream (*n* = 4, 12.1%) infections, and the most common agents were *Escherichia coli* (10 episodes, 30.3%) and *Klebsiella pneumoniae* (6 episodes, 18.2%; Table 1, Fig. 2). *E. coli* was also the most common 3GCR-GN gut coloniser (36/62 3GCR-GN colonisation episodes, 58%), followed by *Enterobacter hormaechei*, *K. pneumoniae, Klebsiella aerogenes,* and *Klebsiella oxytoca* (four episodes, 6.5%, each, Table 1). Thirty-three unique VRE colonisation episodes were identified. Overall, 88 patients were colonised and/or infected with 3GCR-GN and/or VRE. Most (62, 70.5%) experienced a single episode of either 3GCR-GN (*n* = 61) or VRE (*n* = 1), however 17 patients (20.2%) had two episodes (16 patients with two different species/STs, 13 with one VRE and one 3GCR-GN species) and nine patients (10.1%) had ≥3 episodes (seven with ≥3 different species/STs, including five with VRE).

**Table 1:** Colonisation and infection episodes by species

Species	Total colonisation episodes (n baseline)	Infection episodes			
		Total	Pt colonised with same strain (n baseline)	Pt colonised at baseline (%)	Pt not colonised at baseline (%)
**VRE**					
*Enterococcus faecium*	33 (25)	2	0	0	1 (0.4%)
**3GC-resistant GN**					
*Escherichia coli*	36 (33)	10	1 (0)	2 (6%)	4 (2%)
*Klebsiella pneumoniae*	4 (2)	7	3 (2)	2 (100%)	3 (1%)*
*Pseudomonas aeruginosa*	3 (2)	2	1 (1)	2 (100%)	0 (0%)*
*Enterobacter hormaechei*	4 (3)	3	0	0	1
*Klebsiella aerogenes*	4 (1)	3	0	0	1
*Enterobacter asburiae*	0	2	0	0	1
*Stenotrophomonas maltophilia*	0	2	0	0	1
*Serratia marcescens*	1 (0)	2	0	0	0
*Achromobacter xylosoxidans*	0	1	0	0	1
*Burkholderia cenocepacia*	0	1	0	0	1
*Citrobacter freundii*	2 (2)	1	0	0	0
*Citrobacter portucalensis*	1 (1)	0	0	0	0
*Enterobacter kobei*	1 (0)	0	0	0	0
*Escherichia marmotae*	1 (1)	0	0	0	0
*Klebsiella oxytoca*	4 (4)	0	0	0	0
*Morganella morganii*	1 (0)	0	0	0	0

* *p* < 0.00005 for association between colonisation at baseline and infection with same species (proportion test). Distinct episodes of colonisation and infection were defined as those with a unique combination of patient ID, specimen type, species and multi-locus sequence type. Colonisation episodes are expressed as total detected and number detected at baseline (within first 2 days after ICU admission). Strains were defined on the basis of chromosomal SNVs (≤7 SNVs between isolates). *Pt* patient, *VRE* vancomycin-resistant enterococci

The majority of 3GCR-GN colonisation and infection isolates were predicted to be MDR (see Methods): *n* = 44/61 (72%) distinct colonisation episodes and *n* = 18/29 (62%) distinct infection episodes. MDR was particularly common among *E. coli* (*n* = 44 episodes, 96%), *K. pneumoniae* (*n* = 9, 90%) and *Serratia marcescens* (*n* = 3, 100%). WGS identified acquired ESBL genes in 20 representative 3GCR-GN colonisation (32.3%) and 12 representative 3GCR-GN infection (36.4%) isolates. These were mostly MDR *K. pneumoniae* carrying *bla*_CTX-M-15_ (*n* = 8) or MDR *E. coli* carrying diverse *bla*_CTX-M_ genes (*n* = 19, including *n* = 6 *bla*_CTX-M-14_, *n* = 5 *bla*_CTX-M-15_, *n* = 3 *bla*_CTX-M-27_, *n* = 3 *bla*_CTX-M-62_, *n* = 2 *bla*_CTX-M-24-like_). Carbapenemase genes were rare: two patients had wound or tissue infections with *K. pneumoniae* ST231 carrying *bla*_OXA-48,_ one of whom was also colonised by *K. pneumoniae* ST231 with *bla*_OXA-48._ A third patient was colonised with *S. marcescens* carrying *bla*_IMP-4_. One of the patients with *K. pneumoniae* ST231 wound / tissue infections received a course of meropenem (this patient also had 3GCR *P. aeruginosa* and 3GCR *Burkholderia cenocepacia* infections), while the second received meropenem, ciprofloxacin and tigecycline. The *S. marcescens* rectal colonisation episode was not treated.

Most VRE isolates representing distinct episodes (*n* = 32, 91%) carried the *vanB* vancomycin resistance operon and the remainder (*n* = 3, 9%) the *vanA* operon, which confers resistance to vancomycin plus teicoplanin [32]. Additionally, 32 of these isolates (91%) were predicted to be MDR due to the presence of genes conferring resistance to tetracyclines and/or high-level resistance to gentamicin, streptomycin and/or streptogramins.

We assessed species-specific infection rates amongst patients testing culture-positive at baseline screening, versus those testing culture-negative, for all species with ≥2 infections identified amongst patients with baseline screening data (*E. coli, K. pneumoniae, P. aeruginosa*). Infection rates were higher amongst baseline-culture-positive patients, although the difference was statistically significant only for *K. pneumoniae* and *P. aeruginosa* (Table 1). There were few patients for whom WGS data were available for isolates of the same species detected in baseline screening and infections (two patients for each species). For *n* = 2/2 such cases with *K. pneumoniae* (ST231, ST323) and *n* = 1/2 with *P. aeruginosa* (ST357), the WGS data confirmed the infections were caused by the colonising strain (0–7 pairwise SNVs), whereas both *E. coli* infections were caused by different strains to those detected on baseline screening swabs (different STs; Table 1, Supplementary Results, Additional File 1). A further two patients tested culture-negative on baseline screening, but subsequently tested positive on follow-up screening for the same strain that was isolated from their diagnostic specimens (*K. pneumoniae* ST323, *E. coli* ST393; Supplementary Results, Additional File 1).

**Fig. 3:**
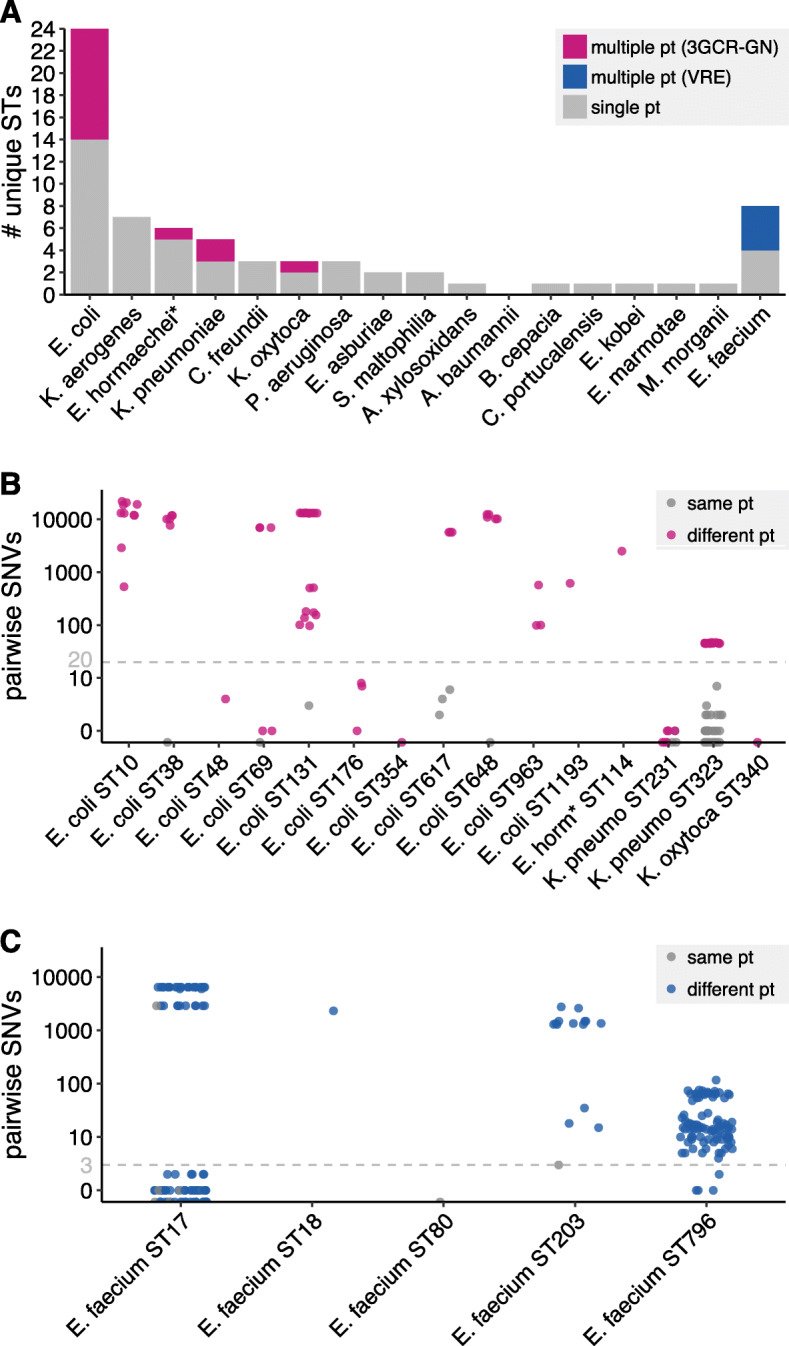
3GCR-GN and VRE strain diversity. (**A**) Bar plot showing the number of unique multi-locus sequence types (STs) identified for each third-generation cephalosporin-resistant Gram-negative (3GCR-GN) species plus vancomycin-resistant *E. faecium* (VRE) Bars are coloured as indicated in the legend. **(B, C)** Pairwise single nucleotide variants (SNVs) for pairs of isolates assigned to the same ST for 3GCR-GN species (**B**) and VRE (**C**). Grey points indicate pairs of isolates from the same patient (pt), pink and blue points indicate pairs of isolates from different patients for 3GCR-GN and VRE isolates, respectively. Grey dashed lines indicate the SNV thresholds used to define putative transmissions (*n* ≤ 20 SNVs for 3GCR-GN and *n* ≤ 3 SNVs for VRE, see Results for details). *E. hormaechei multi-locus sequence typing was performed using the *Enterobacter cloacae* scheme, which covers both species

Finally, we assessed strain level diversity and evidence for nosocomial transmission using WGS and patient data. The isolates were diverse, with 62 STs identified amongst 3GCR-GN (24 amongst *E. coli* alone, including *n* = 6 ST131 episodes, *n* = 4 ST10 and *n* = 3 episodes each of ST38, ST69, ST176, ST648 and ST963) and 8 amongst VRE (including *n* = 14 ST796, *n* = 10 ST17 and *n* = 5 ST203 episodes). Most 3GCR-GN STs (*n* = 48, 77%) were unique to a single patient; however, ten *E. coli* STs (42%), two *K. pneumoniae* (40%), one *K. oyxtoca* (33%), one *Enterobacter hormachaei* (17%) and five VRE STs (63%) were found in multiple patients (Fig. 3a). We defined probable strain transmission events on the basis of chromosomal SNVs (see Methods, Fig. 3b-c). Using a threshold of ≤20 SNVs for transmission of 3GCR-GN (based on recent studies [10, 15, 16, 33]) we identified six putative 3GCR-GN transmission clusters each involving 2–3 patients (maximum 8 SNVs, Table 2). With a single exception, all patients within clusters were epidemiologically linked (overlapping ICU stays or ≤ 14 days between stays). Assuming one patient in each epidemiologically linked cluster was the index patient, the data suggest only six (6.3%) 3GCR-GN episodes resulted from recent nosocomial transmission (*n* = 2, 6.1% of infections; *n* = 4, 6.5% of colonisation). We also identified *K. pneumoniae* ST323 isolates from two patients that differed by 45–47 SNVs, notably many fewer than the majority of comparisons (Fig. 3a) and within the range expected for *K. pneumoniae* subject to wider circulation in the healthcare network rather than those subject to community-transmission [33]. Consistent with this, we have previously shown that *K. pneumoniae* ST323 were circulating more broadly within the hospital during the study period [34].

**Table 2:** Putative nosocomial transmission clusters

#	Organism	Cluster size (n pt)		Pairwise SNVs	Days between pt. stays	Episodes attributed to transmission (n total (n very likely)) ^c^
		Genomic ^a^	Genomic + epi ^b^			
1	*E. coli* ST48	2	2	4	8	1 (0)
2	*E. coli* ST69	2	2	1	0	1 (1)
3	*E. coli* ST176	3	2	1–8	0–31	1 (1)
4	*E. coli* ST354	2	2	0	6	1 (0)
5	*K. pneumoniae* ST231	2	2	0–2	0	1 (1)
6	*K. oxytoca* ST340	2	2	0	0	1 (1)
7	*E. faecium* ST17	2	2	1	11	1 (0)
8	*E. faecium* ST17	7	7	0–2	0	6 (6)
9	*E. faecium* ST796	2	2	1	1	1 (0)
10	*E. faecium* ST796	3	0	1–2	20–62	0 (0)

Pt, patient; SNVs, single nucleotide variants

^a^Patient cluster identified by genomic analysis; ≤ 20 single nucleotide variants between isolates (Gram-negative species), or ≤ 3 single nucleotide variants between isolates (*E. faecium*)

^b^Patient cluster identified by genomic analysis (as defined above) and supported by epidemiological data (≤ 14 days between patient ICU stays or overlapping stays)

^c^Number of colonisation and infection episodes likely or very likely attributed to transmission; likely, patient was part of the genomic cluster (as defined above) with ≤14 days between the patient ICU stay and that of another patient in the genomic cluster; very likely, patient was part of the genomic cluster (as defined above) and ICU stay overlapped with ≥1 other patient in the genomic cluster

Interpretation of VRE pairwise SNVs is more complex because several healthcare-associated lineages are known to be circulating in Melbourne and the regional hospital network. Consequently, it is not uncommon to find near-identical isolates in patients from different wards, hospitals or cities without epidemiological links [7, 8, 15]. We therefore used a more conservative threshold of ≤3 SNVs to define putative recent VRE transmissions (based on empirical distribution, see Supplementary Figure 3), which identified four clusters of 2–7 patients each (Table 2). Two clusters were ST17 (both epidemiologically linked clusters, separated by ≥2900 SNVs) and two were the locally emerged ST796 [35, 36] (clusters separated by 4–6 SNVs, one was epidemiologically linked). Assuming one patient in each epidemiologically linked cluster is the index patient, we estimate eight (28.6%) VRE episodes resulted from recent transmission (*n* = 1 infection and *n* = 7, 21.2% of colonisation episodes). This should be considered a conservative estimate considering the difficulty in distinguishing clusters, particularly for ST796. Nevertheless, the transmission burden is significantly greater than that estimated for 3GCR-GN organisms (OR = 4.3, 95% CI 1.2–16.6, *p* = 0.02 using Fisher’s Exact test).

## Discussion

We have presented a holistic investigation of 3GCR-GN and VRE colonising and infecting patients in an Australian ICU over a three-month duration. Approximately 17% patients were colonised by at least one 3GCR-GN at ICU admission. Just under 40% of these isolates were detected as ESBL-*Enterobacteriaceae,* hence our data are comparable to reports from Europe where 7–13% of ICU-admitted patients were colonised with these organisms [17, 19, 37], and indicate a lower prevalence of ESBL-*Enterobacteriaceae* colonisation than reported among patients admitted to ICUs in China (32%) [38] and Thailand (62%) [39]. Additionally, 9% of patients in our study were colonised with VRE at ICU admission; a similar prevalence to that reported recently for patients in a hospital network in Singapore [40], higher than that reported for patients admitted to an ICU in Sri Lanka (2%) [41], and lower than reported in Brazil (15%) [42] and India (31%) [43]. Notably, the VRE colonisation prevalence was lower than the 17.5% (95% CI 13.7–21.9) indicated in a previous point-prevalence survey of patients in our hospital [44].

*E. coli* was the dominant colonising and infecting organism, and has been previously reported as the most common cause of GN blood-stream infections in Australia [5]. These findings are consistent with the notion of colonisation as a prerequisite for disease and the logic of scales, whereby the most common colonisers have more opportunities to cause infection and therefore contribute the greatest burden of disease. Our data indicated a higher prevalence of infections among patients colonised at admission with 3GCR *E. coli*, *K. pneumoniae* or *P. aeruginosa* than those who were not colonised. However, there were differences in the rate of infection; just 6% for *E. coli*, versus 100% (*n* = 2/2 each) for *K. pneumoniae* and *P. aeruginosa*. While these data must be interpreted with caution due to the small sample size, we note that the trends are consistent with previous reports [9, 17] including a large study of 2386 patients colonised by ESBL-*K. pneumoniae* or ESBL-*E. coli,* which found that the former were twice as likely as the latter to develop an infection with the same organism (6.8% versus 3.2% [9]).

At the time of this study our hospital infection prevention and control (IPC) policy stated that all patients known to have a clinical VRE and/or carbapenemase-producing *Enterobacteriaceae* (metallo-beta-lactamases only) infection or colonisation were isolated and subject to contact precautions during their hospital admission. Nevertheless, our WGS analysis identified four putative VRE transmission clusters (including three that were epidemiologically linked) and indicated that 21.2% of VRE episodes were attributable to transmission. In contrast, we estimated that just 6.3% of 3GCR-GN episodes were attributable to transmission (21.2% VRE vs 6.3% 3GCR-GN, OR 4.3, *p* = 0.0209), resulting from six putative 3GCR-GN transmission clusters (all epidemiologically linked). However, no specific IPC policies were applied for patients known to be colonised or infected with 3GCR-GN organisms beyond standard care.

The putative VRE transmission clusters involved ST17 and ST796, which are considered healthcare-adapted lineages and are widely distributed in Australia [5–8, 36, 45]. While four of the six 3GCR-GN transmission clusters involved *E. coli*, these included just four of 24 distinct *E. coli* STs, and did not include the globally distributed ST131, ST10 or ST38. This is consistent with a recent report showing that these *E. coli* STs were commonly identified from patients in other Melbourne hospitals, but rarely associated with nosocomial transmission [15]. Hence our data adds to the growing evidence base [15, 18–20, 46] that ESBL-*E. coli* are mainly spread in the community setting.

While the risk of ESBL-*E. coli* transmission in hospitals may be low, there is emerging evidence that the risk may be 2–4 times greater for other ESBL-*Enterobacteriaceae* such as *K. pneumoniae* and *Enterobacter* sp. [19, 20]. In our study, 2/11 (18.2%) ESBL-*K. pneumoniae* episodes were attributed to recent transmission, with one additional episode attributed to broader transmission within the hospital as reported previously [34]; however, a larger sample size is required to make a definitive comparison.

The inclusion of contemporaneous rectal screening and clinical isolates is a key strength of this study, which in combination with the breadth of species sampling and use of WGS, has allowed high-resolution analysis of the dynamics of AMR organisms in the ICU. Notably, the majority of putative transmissions we identified would have been missed if not for the inclusion of rectal screening. However, the study also has several limitations: firstly, we were not able to screen all eligible patients, nor to follow our patients beyond the three-month study period, thus we likely underestimate the true burdens of infection and transmission. Secondly, due to its short duration this study was underpowered for the assessment of patient risk factors (e.g. recent surgery or antibiotic use), which we expect to impact the risk of infection and onwards transmission. Thirdly, our focus on the ICU as a high-risk setting for HAI fails to capture the additional burden of 3GCR-GN and VRE in other wards. Finally, as noted above our assessment of infection risk among patients colonised with 3GCR-GN species other than *E. coli* was limited due to sample size. Nevertheless, in combination with the small number of existing reports our results highlight and contrast several key features of 3GCR-GN and VRE dynamics, and the importance of understanding species-specific risk(s).

## Conclusions

Our data show that 3GCR-GN and VRE causing gut colonisation and infections in the ICU are highly diverse, and that the most common AMR colonisers (VRE and *E. coli*) do not necessarily pose the greatest risk to patients (in terms of incident infection, or onward transmission) in comparison to other gut colonising species. A more detailed assessment of species-specific risks (especially attack rate and risk of transmission) is needed to guide the efficient use of rectal screening programs and targeted approaches to IPC aimed at limiting the MDR disease burden.

## Supplementary Information

**Additional file 1.** Supplementary Methods, Supplementary Results, Supplementary Tables 2-6, Supplementary Figures 1-3.

**Additional file 2: Supplementary Table 1.** Specimen and genotype information of isolates included in this study.

## Data Availability

The datasets supporting the conclusions of this article are included within the article (and its additional files and/or available via public data repositories: Bacterial isolate metadata and genotypes are listed in Supplementary Table 1, Additional File 2. Illumina read data are publicly available via the European Nucleotide Archive (https://www.ebi.ac.uk/ena) under project accessions PRJEB6891 and PRJNA646837, and hybrid genome assemblies are publicly available via GenBank (https://www.ncbi.nlm.nih.gov/genbank/); accessions are listed in Supplementary Table 1, Additional File 2.

## Abbreviations

ICU: Intensive care unit
IPC: Infection prevention and control
MDR: Multi-drug resistant
AMR: Antimicrobial resistant
3GCR: Third generation cephalosporin resistant
GN: Gram-negative
VRE: Vancomycin resistant enterococci
ST: Sequence type
SNV: Single nucleotide variant
