# Supplementary material for: Genomic surveillance of antimicrobial resistant bacterial colonisation and infection in intensive care patients
Source: BMC Infect Dis. 2021 Jul 14;21:683. doi: 10.1186/s12879-021-06386-z (PMC8278603; doi:10.1186/s12879-021-06386-z)
Supplement: Supplementary file 1 — Additional file 1. Supplementary Methods, Supplementary Results, Supplementary Tables 2-6, Supplementary Figures 1-3. [file 12879_2021_6386_MOESM1_ESM.docx]

# Genomic surveillance of antimicrobial resistant bacterial colonisation and infection in intensive care patients: Additional File 1

Kelly L. Wyres*^†1^, Jane Hawkey*^1^, Mirianne Mirčeta^2^, Louise M. Judd^1^, Ryan R. Wick^1^, Claire L. Gorrie^3^, Nigel F. Pratt^4^, Jill S. Garlick^4^, Kerrie M. Watson^4^, David V. Pilcher^5,6^, Steve A. McGloughlin^5,6^, Iain J. Abbott^1,7^, Nenad Macesic^1,7^, Denis W. Spelman^7^, Adam W. J. Jenney^7^ and Kathryn E. Holt^1,8^

^1^ Department of Infectious Diseases, Central Clinical School, Monash University, Victoria, Australia

^2^ Microbiology Unit, Alfred Health, Melbourne, Victoria, Australia

^3^ Department of Microbiology and Immunology, University of Melbourne, Victoria, Australia

^4^ Infectious Diseases Clinical Research Unit, The Alfred Hospital, Melbourne, Victoria, Australia

^5^ Intensive Care Unit, The Alfred Hospital, Melbourne, Victoria, Australia

^6^ Australian and New Zealand Intensive Care – Research Centre, School of Public Health and Preventive Medicine, Monash University, Victoria, Australia

^7^ Department of Infectious Diseases, The Alfred Hospital, Melbourne, Victoria, Australia

^8^ London School of Hygiene and Tropical Medicine, London, United Kingdom

* These authors contributed equally to this work.

Corresponding authors details: Kelly Wyres; Department of Infectious Diseases, Central Clinical School, Monash University, Victoria, Australia; kelly.wyres@monash.edu

Adam Jenney; Microbiology Unit & Department of Infectious Diseases, The Alfred Hospital, Melbourne, Victoria, Australia; a.jenney@alfred.org.au

Supplementary Methods

#### Bacterial culture and sequencing

Rectal swabs were plated onto ceftazidime agar plates to identify third-generation cephalosporin-resistant Gram-negative (3GCR-GN) organisms and chromID VRE agar to identify vancomycin-resistant enterococci (VRE) (BioMérieux, Marcy L’Etoile, France). Additionally, ciprofloxacin agar plates were used to determine if the Gram-negative organisms were resistant to fluoroquinolones. Plates were incubated in air at 36^o^C for 24-48 hours. Following species identification, purified cultures were stocked in glycerol and stored at -80°C. Clinical isolates were collected and identified via standard diagnostic protocols. The relevant growth media, conditions and quality control strains are listed in **Supplementary Table 5, Additional File 1**. Biochemical identification tests and quality control strains are listed in **Supplementary Table 6, Additional File 1**.

Genomic DNA was extracted from overnight cultures using a phenol:chloroform protocol and phase lock gel tubes as previously described [1]. Paired-end sequencing libraries were prepared using the Nextera XT kit (Illumina Inc) and sequenced on either the Illumina HiSeq platform (3GCR-GN isolates, 125 bp paired-end reads) or the Illumina MiSeq platform (VRE isolates, 300 bp paired-end reads). Isolates representing multi-locus sequence types of interest (those identified from ≥2 patients, or for which gut colonising and infecting isolates were identified from the same patient) were selected for sequencing on the Oxford Nanopore MinION R9.4.1 flow cell (n = 23 isolates, one for each sequence type of interest, see **Supplementary Table 1, Additional File 2**) [2].

#### Genome assembly and quality control

Draft genomes were assembled using SPAdes v3.12.0 [3] optimised in Unicycler v0.4.7 [4] (using default parameters, and separately using the “--depth_filter 0.0” to allow for assessment of contamination). Illumina data were subjected to quality control to exclude low quality genomes if any of the following thresholds were met: 1) estimated depth <20x; 2) total assembly length (from default parameters) >9 Mbp or 3.5 Mbp for 3GCR-GN and VRE, respectively; 3) total assembly length (from default parameters) >6.5 Mbp or 3 Mbp and total contig count >500 for 3GCR-GN and VRE, respectively; 4) total number of assembly graph dead-ends >10 or >110 for 3GCR-GN and VRE, respectively and combined length of contaminant contigs >0.5% genome length; 5) combined length of contaminant contigs >0.5% genome length and contaminants identified as the same species as the dominating organism using Kraken v1.0 [5] (in such a case the contaminants were considered likely to impact downstream mapping and variant-calling analyses, whereas contaminants originating from a different species were unlikely to map to the reference chromosome). Contaminant contigs were identified from the “--depth_filter 0.0” assembly (defined as mean contig depth <0.25x chromosomal depth) and these were filtered out of the default-parameter assemblies that were used for downstream analyses.

For 23 isolates subjected to additional long-read sequencing, completed genome assemblies were generated using Unicycler v0.4.7 [4]. Two VRE assemblies failed to resolve the chromosome into circular replicons via this method, and were assembled independently using Flye v2.6 [6] to perform long-read-only assemblies that were polished first with the Nanopore reads using Medaka (https://github.com/nanoporetech/medaka), then with the Illumina reads using Pilon v1.22 [7]. Genome assemblies have been deposited in Genbank (accession numbers listed in **Supplementary Table 1, Additional File 2**) and are available via Figshare at https://doi.org/10.26180/c.5194736.

Antimicrobial resistance profiles

Antimicrobial resistance (AMR) genes were identified from genome assemblies using Kleborate (github/katholt/kleborate) which employs a curated version of the CARD AMR gene database v3.0.8 [8]. These data were manually interpreted in a species-specific manner (i.e. to distinguish intrinsic from acquired genes) and combined with phenotype data based on the ceftazidime and ciprofloxacin plate growth (3GCR-GN only) or chromID VRE plate growth (VRE only) to calculate the predicted number of acquired AMR classes per isolate, defined on a species-specific basis as described in [9] (acquired resistances could not be determined for six isolates for which species definitions were unavailable, see **Table S1**). High-level resistances for gentamicin, streptomycin and streptogramins were predicted for *E. faecium* genomes on the basis of the relevant acquired genes described in [10].

Supplementary Results

Comparison of colonising and infecting isolates from the same patient

We identified patients who had infection and colonisation episodes caused by the same species and sequence type (ST). Overall, four of 36 patients colonised by *E. coli* (11.1%) also had an *E. coli* infection but only one of these was caused by an isolate sharing the same ST as the colonising strain (2.7%). Similarly, one of 33 patients colonised by VRE had a VRE infection and this was caused by an isolate of the same ST (3.0%). In contrast, three of four patients colonised by *K. pneumoniae* (75.0%) and two of three patients colonised by *P. aeruginosa* (66.6%) also had infections caused by isolates sharing the same ST as the colonising strain.

The groups of matching colonisation and infection isolates comprised *E. coli* ST393, *K. pneumoniae* ST323 (two patients), *K. pneumoniae* ST231, *P. aeruginosa* ST357, *P. aeruginosa* ST471 and VRE ST17. We generated high quality completed reference genomes for one representative isolate of each of these STs (see **Supplementary Table 1, Additional File 2**). We then compared all available isolates from each of the case patients to their respective ST reference genomes, which confirmed that the *E. coli,* the *P. aeruginosa* ST357 and the three *K. pneumoniae* infections were caused by strains that were very closely related to those isolated from rectal carriage in the same patient (0, 0 and 0-7 chromosomal SNVs between pairs of infection and carriage isolates, respectively). These included the two *K. pneumoniae* infections (one ST231 and one ST323) and the *P. aeruginosa* infection (ST357) reported in **Results** among patients colonised by the same strain at baseline. The second patient harbouring *K. pneumoniae* ST323 was negative for rectal colonisation at base-line screening, but was positive from a rectal swab and a sputum sample collected 39 days later. The patient carrying *E. coli* ST393 was negative for rectal colonisation at baseline but positive from a subsequent rectal swab collected after a positive blood culture. The pair of VRE ST17 isolates from patient AH0390 (one from a rectal swab collected >2 days after admission and one causing empyema) differed by 2904 SNVs, suggesting that these were unrelated episodes. Supporting this hypothesis, our subsequent transmission analyses indicated that the infecting isolate was part of a transmission cluster and was not the index case, whereas the carriage isolate was not part of any transmission cluster. Comparison of the two *P. aeruginosa* ST471 isolates from patient AH0296 (one baseline rectal carriage and one respiratory infection isolate) suggested that these were independent (458 SNVs). Review of the patient record indicated that AH0296 was a cystic fibrosis patient with chronic *Pseudomonas* sp. colonisation; hence it is possible that the rectal carriage and sputum isolates represent divergent descendants from a single common ancestor that was acquired months or years prior to this study.

References

1. Gorrie CL, Wick RR, Edwards DJ, et al. Gastrointestinal carriage is a major reservoir of *K. pneumoniae* infection in intensive care patients. Clin Infect Dis **2017**; 65:208–215.

2. Wick RR, Judd LM, Gorrie CL, Holt KE. Completing bacterial genome assemblies with multiplex MinION sequencing. MGen **2017**; 3.

3. Bankevich A, Nurk S, Antipov D, et al. SPAdes: a new genome assembly algorithm and its applications to single-cell sequencing. J Comput Biol **2012**; 19:455–477.

4. Wick RR, Judd LM, Gorrie CL, Holt KE. Unicycler: resolving bacterial genome assemblies from short and long sequencing reads. PLoS Comp Biol **2017**; 13:e1005595.

5. Wood DE, Salzberg SL. Kraken: Ultrafast metagenomic sequence classification using exact alignments. Genome Biol **2014**; 15:R46.

6. Kolmogorov M, Yuan J, Lin Y, Pevzner PA. Assembly of long, error-prone reads using repeat graphs. Nat Biotechnol **2019**; 37:540–546.

7. Walker BJ, Abeel T, Shea T, et al. Pilon: An integrated tool for comprehensive microbial variant detection and genome assembly improvement. PLoS One **2014**; 9:e112963.

8. Alcock BP, Raphenya AR, Lau TTY, et al. CARD 2020: Antibiotic resistome surveillance with the comprehensive antibiotic resistance database. Nucleic Acids Res **2020**; 48:D517–D525.

9. Magiorakos A, Srinivasan A, Carey RB, et al. Multidrug-resistant, extensively drug-resistant and pandrug-resistant bacteria: an international expert proposal for interim standard definitions for acquired resistance. Clin Microbiol Infect **2012**; 18:268–281.

10. Hollenbeck BL, Rice LB. Intrinsic and acquired resistance mechanisms in enterococcus. Virulence **2012**; 3:421–569.

Supplementary Tables

**Supplementary Table 1: Specimen and genotype information of isolates included in this study.** See separate Excel sheet in **Additional File 2**, also available at https://doi.org/10.26180/c.5194736.

**Supplementary Table 2: Characteristics of the patient cohort subjected to rectal screening within the first two days of ICU admission**

|  | **N Participants (%)** |
| --- | --- |
| Sex (male) | 185 (64.5) |
| Age (years): |  |
| 18-29 | 32 (11.2) |
| 30-39 | 27 (9.4) |
| 40-49 | 36 (12.5) |
| 50-59 | 62 (21.6) |
| 60-69 | 49 (17.1) |
| 70-79 | 52 (18.1) |
| >80 | 29 (10.1) |
| Antibiotics last 7 days | 219 (76.3) |
| Surgery last 30 days | 177 (61.7) |
| Recent healthcare exposure^a^ | 218 (76.0) |
| **Total** | 287 |

^a^ surgical procedure within the last 30 days, transferred from another ward in the Alfred hospital with first admission >2 days prior and/or transferred from another hospital.

**Supplementary Table 3: Logistic regression for factors associated with colonisation (culture-positive rectal swab) at baseline screening (day 0-2 of ICU admission)**

|  | **N +ve (%)** | **N -ve (%)** | **p-value** |
| --- | --- | --- | --- |
| **3GCR-GN** | **50 (17)** | **237 (83)** |  |
| **Univariate analysis** |  |  |  |
| Sex (male) | 36 (19) | 149 (81) | 0.42 (p=0.22) |
| Age | na | na | -0.01 (p=0.41) |
| Age (within males) | na | na | -0.01 (p=0.38) |
| Age (within females) | na | na | -0.00 (p=0.85) |
| Antibiotics last 7 days | 42 (19) | 177 (81) | 0.58 (p=0.16) |
| Surgery last 30 days | 28 (16) | 149 (84) | -0.29 (p=0.37) |
| Recent healthcare exposure^a^ | 37 (17) | 181 (83) | -0.13 (p=0.72) |
| **Multivariate analysis** |  |  |  |
| Sex (male) | 36 (19) | 149 (81) | 0.48 (p=0.17) |
| Age (years) | na | na | -0.01 (p=0.54) |
| Antibiotics last 7 days | 42 (19) | 177 (81) | 0.77 (p=0.08) |
| Surgery last 30 days | 28 (16) | 149 (84) | -0.51 (p=0.13) |
| **VRE** | **24 (8)** | **263 (92)** |  |
| **Univariate analysis** |  |  |  |
| Sex (male) | 16 (9) | 169 (91) | 0.11 (p=0.81) |
| Age | na | na | 0.03 (p=0.04)* |
| Age (within males) | na | na | 0.02 (p=0.31) |
| Age (within females) | na | na | 0.04 (p=0.05) |
| Antibiotics last 7 days | 20 (9) | 199 (91) | 0.48 (p=0.40) |
| Surgery last 30 days | 16 (9) | 161 (91) | 0.24 (p=0.60) |
| Recent healthcare exposure^a^ | 21 (10) | 197 (90) | 0.85 (p=0.18) |
| **Multivariate analysis** |  |  |  |
| Sex (male) | 16 (9) | 169 (91) | 0.14 (p=0.77) |
| Age (years) | na | na | 0.03 (p=0.04)* |
| Antibiotics last 7 days | 20 (9) | 199 (91) | 0.50 (p=0.41) |
| Surgery last 30 days | 16 (9) | 161 (91) | -0.00 (p=1.00) |

^a^ surgical procedure within the last 30 days, transferred from another ward in the Alfred hospital with first admission >2 days prior and/or transferred from another hospital.

N +ve, number of patients culture positive at baseline (within row category); N -ve, number of patients culture negative at baseline (within row category); 3GCR-GN, third-generation cephalosporin-resistant Gram-negative; VRE, vancomycin resistant enterococci; *, *p*≤0.05; ** *p*<0.0005.

**Supplementary Table 4: Prevalence of third generation cephalosporin-resistant Gram-negatives (3GCR-GN) and vancomycin-resistant *E. faecium* (VRE) isolated from rectal screening swabs**

| Time since admission (days) | Swabs processed  (n) | VRE positive  (n) | VRE prevalence (%) | 3GCR-GN positive  (n) | 3GCR-GN prevalence (%) | Total GN organisms (n) |
| --- | --- | --- | --- | --- | --- | --- |
| 0-2 | 287 | 24 | 8.4 | 50 | 17.4 | 63 |
| 3-7 | 84 | 6 | 7.1 | 8 | 9.5 | 10 |
| 7-14 | 28 | 2 | 7.1 | 7 | 25.0 | 8 |
| 15-21 | 12 | 3 | 25.0 | 2 | 16.7 | 1 |
| 22-78 | 14 | 4 | 28.6 | 8 | 57.1 | 11 |

**Supplementary Table 5: Growth media, conditions and quality control strains for bacterial species identification**

| **Medium and conditions ^a^** | **Control organisms ^b^** | **Expected result** |
| --- | --- | --- |
| Horse blood agar,  CM331  (pH 7.3 + 0.2), CO_2_, 24 hrs, 35^o^C | *Streptococcus pyogenes* ATCC 19615  (1:100)  *Streptococcus pneumoniae* ATCC 6305  (1:100)  *Haemophilus influenzae* ATCC 10211  (1:100) with *Staphylococcus* streak | Growth (β haemolysis)  Growth (α haemolysis)  Growth (satellitism) |
| Horse blood agar,  CM331  (pH 7.3 + 0.2), AnO_2_, 24 hrs, 35^o^C | *Bacillus fragilis* ATCC 25285 (1:100)  *Clostridium perfringens* ATCC 13124 (1:100) | Growth (colonies 2–3 mm)  Growth (haemolysis) |
| MacConkey agar no.3, CM115  **(**pH 7.1 + 0.2), O_2_, 24 hrs, 35^o^C | *Escherichia coli* ATCC 25922 (1:100)  *Salmonella enterica* Typhimurium ATCC 14028 (1:100)  *Proteus mirabilis* ATCC 12453 (1:100) | Growth (lactose +ve pink colonies 2-3mm)  Growth (clear colonies 2-3mm)  Growth (lactose -ve clear clonies 2-3mm, no swarming) |
| *Burkholderia cepacia* selective agar, O_2_, 120 hrs,  35^o^C | *B. cepacia* ATCC 25608 (1:100)  *P. aeruginosa* ATCC 27853 (1:10) | Growth (mauve / grey colonies)  No growth |
| Chrom/CNA split agar (pH 7.3 + 0.2), O_2_, 24 hrs, 35^o^C | **Chrom**  *E. coli* ATCC 25922 (1:100)  *P. mirabilis* ATCC 12453 (1:100)  *Staphylococcus aureus* ATCC 25923 1:100)  *Enterococcus faecalis* ATCC 29212 (1:100)  **CNA**  *E. coli* ATCC 25922 (1:10)  *P. mirabilis* ATCC 12453 (1:10)  *S. aureus* ATCC 25923 (1:100)  *E. faecalis* ATCC 29212 (1:100) | Growth (pink colonies)  Growth (orange-brown colonies)  Growth (white colonies)  Growth (turquoise-blue colonies)  No growth  No growth  Growth  Growth |
| VRE chromogenic medium, O_2_, 72 hrs, 35^o^C | *E. faecalis* ATCC 29212 (1:10)  *E. faecalis* 305-2 (Van B+) QC 3 (1:10)  *Enterococcus faecium* (Van B+) Qc10 (1:10) | No growth  Growth (blue-green colonies)  Growth (violet colonies) |

^a^ atmosphere, length of incubation and temperature. ^b^ numbers in parentheses indicate dilution of 0.5 MacFarland.

**Supplementary Table 6: Biochemical tests and quality control strains for bacterial species identification**

| **Reagent / test ^a^** | **Strain** | **Expected result** |
| --- | --- | --- |
| API20E | *Proteus mirabilis* ATCC 35659 | Profile 0736000 |
| APICoryne | *Corynebacterium renale* ATCC 19412 | Profile 2201304 |
| API20NE | *Aeromonas hydrophilia* ATCC 35654  *Alcaligenes faecalis* ATCC 35655 | Profile 7577754  Profile 0000057 |
| APIStrep | *Streptococcus equi* ssp *zooepidemicus* ATCC 700400 | Profile 016 607 |
| Bile solubility | *Streptococcus pneumoniae* ATCC 6305 | Bile soluble |
| Catalase | *Staphylococcus aureus* ATCC 25923  *Enterococcus faecalis* ATCC 29212 | Positive  Negative |
| Catarrhalis test discs | *Moraxella catarrhalis* ATCC 25238  *Neisseria lactamica* ATCC 23970 | Blue green colour change  No colour change |
| Coagulase  (rabbit plasma) | *S. aureus* ATCC 25923  *Staphylococcus epidermidis* ATCC 14990 | Positive  Negative |
| Cryptococcal antigen | Clinical sample CSF | Positive (titre as per individual clinical sample) |
| *Clostridium difficile* latex | *C. difficile* ATCC 43593 | Positive |
| Germ tube  (rabbit plasma) | *Candida albicans* ATCC 60193  *Candida glabrata* QC 1 | Positive  Negative |
| Indole (spot) | *Escherichia coli* ATCC 25922  *P. mirabilis* ATCC 12453 | Positive  Negative |
| Legionella Urinary Antigen | Positive control swab | Positive |
| Metronidazole disc | *Bacillus fragilis* ATCC 25285 | Zone of inhibition |
| Oxidase (spot) | *Pseudomonas aeruginosa* ATCC 27853  *E. coli* ATCC 25922 | Positive  Negative |
| Optochin | *S. pneumoniae* ATCC 49619  *E. faecalis* ATCC 29212 | Sensitive  Resistant |
| Pyrrolidonyl aminopeptidase | *E. faecalis* ATCC 29212  *Streptococcus agalactiae* ATCC 13813 | Positive  Negative |

^a^ API strips were obtained from BioMérieux, Marcy L’Etoile, France.

Supplementary Figures


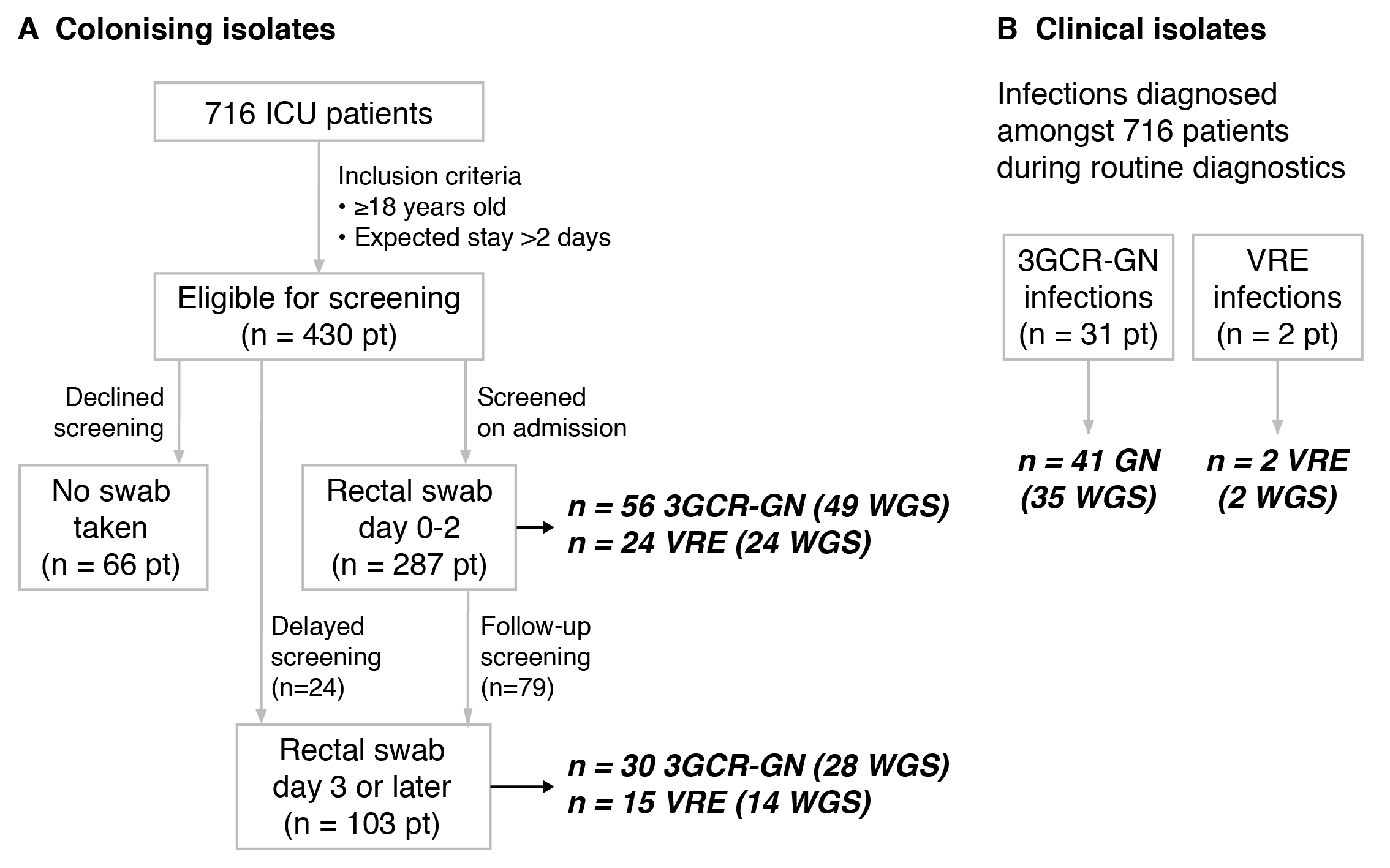


**Supplementary Figure 1: Sources of (A) colonising and (B) clinical isolates from ICU patients.** 3GCR-GN, third generation cephalosporin-resistant Gram-negatives; VRE, vancomycin-resistant enterococci; pt patients; WGS, whole-genome sequences. Bold italics indicates number of isolates, numbers in parentheses indicate the number of WGS included in analyses.


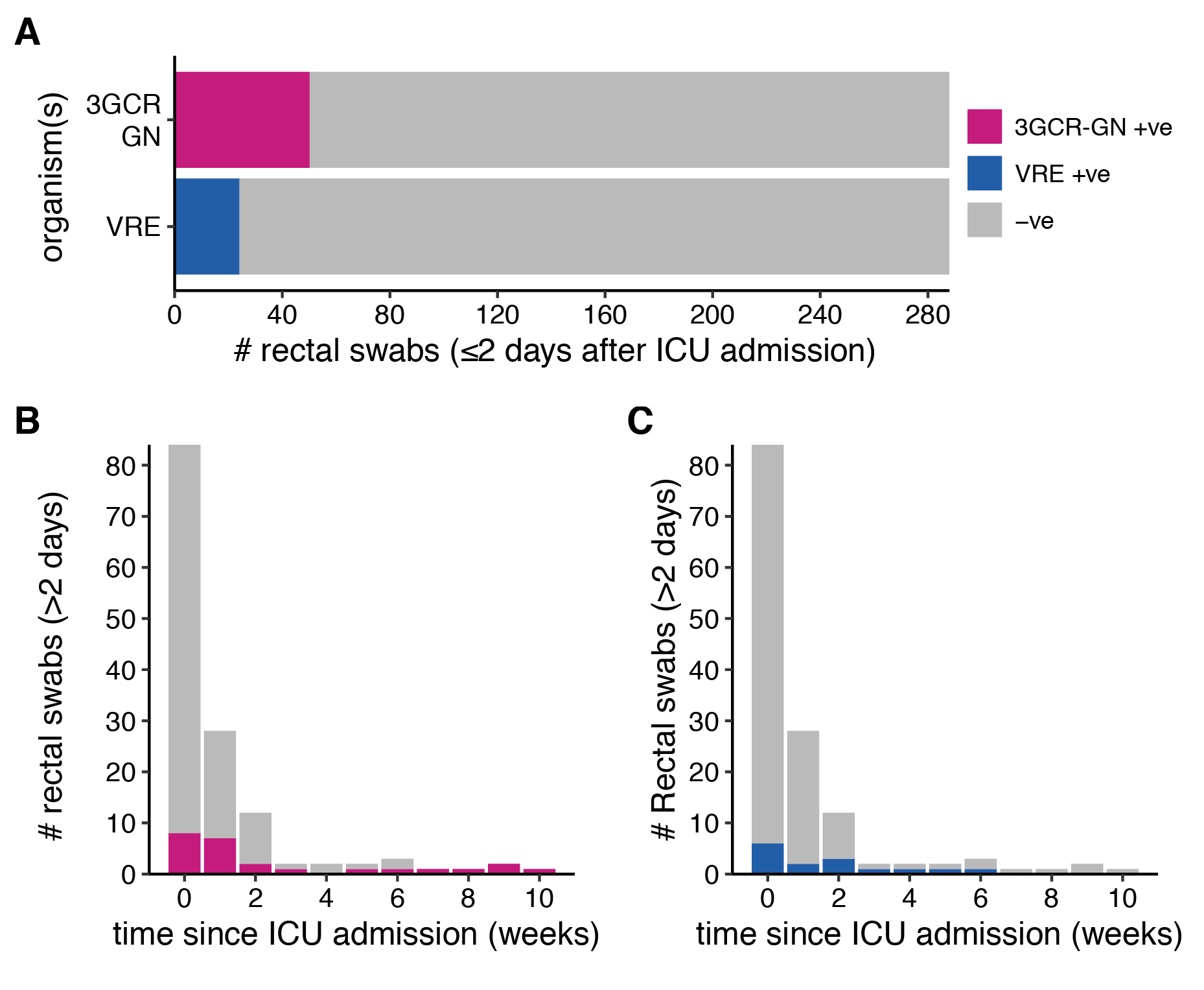


**Supplementary Figure 2: Prevalence of third generation cephalosporin-resistant Gram-negative (3GCR-GN) and vancomycin resistant enterococci (VRE) gut colonisation stratified by time since ICU admission.** (**A**) Prevalence of 3GCR-GN organisms and VRE among patients swabbed within the first two days of ICU admission (baseline swabs). Prevalence of 3GCR-GN (**B**) and VRE (**C**) among patients, for swabs collected >2 days after ICU admission.


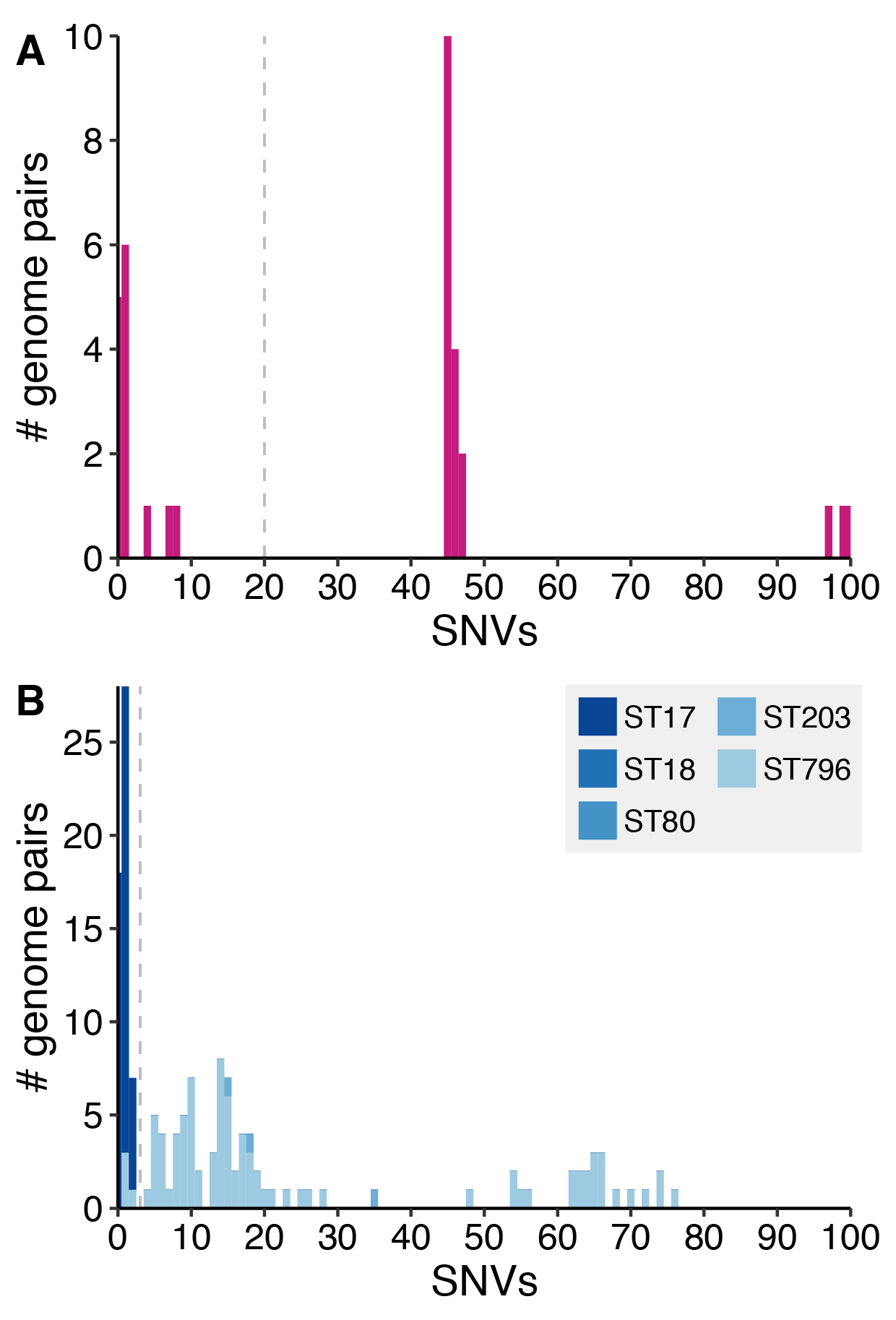


**Supplementary Figure 3: Distribution of single nucleotide variant (SNV) counts ≤100 for pairs of (A) third-generation cephalosporin-resistant Gram-negative (3GCR-GN) and (B) vancomycin resistant enterococci (VRE) isolates from different patients.** Grey dashed lines indicate the SNV thresholds used to define putative transmissions (n ≤ 20 SNVs for 3GCR-GN and n ≤ 3 SNVs for VRE). Bars in (**B**) are coloured by the *E. faecium* multi-locus sequence type (ST) of the isolates as indicated in the legend.
